# The Book World of Medicine and Science

**Published:** 1906-06-09

**Authors:** 


### The Book World of Medicine and Science.

New Serum Therapy. By D. Montgomerie Paton, L.R.C.S., L.R.C.P. (Baillière, Tindall, and Cox.)

The writer of this book maintains, as the result of experience, that normal plasma of some animals given to debilitated subjects stimulates their nutrition, and aids the normal power of resistance against disease possessed by their tissues. Dr. Montgomerie states that one drachm of horse or sheep plasma given four times a day not only to be of great value in anæmia, but also in septic infections. The illustrations given of its value do not appear to be very conclusive, but it is difficult to say that plasma given in the way suggested does not have any beneficial effect. It at least is a harmless remedy, and can be tried.

International Clinics. Vol. IV. Fifteenth series. Edited by A. O. J. Kelly, M.D. (Philadelphia and London: J. B. Lippincott Company. 1906. Pp. 312.)

It is difficult to do justice in the space of a short review to a volume such as this one, containing many papers on various subjects by different authors. The "International Clinics" are now widely known and appreciated, and it is not necessary to offer further commendation than to say that the present volume is rather above than below the high average standard of its predecessors. Most of the contributing authors are American. The book contains, among many others, papers on the following subjects: The treatment of psoriasis, the therapeutic value and the mode of action of normal saline solution, the internal use of carbolic acid, the later stages of cirrhosis of the liver, the diagnosis of Malta fever, the results of operations on the stomach, phlebitis, thrombosis and embolism following abdominal and pelvic operations, the study of the clinical course of joint tuberculosis by means of the $x$-rays. We mention the titles of some of the papers because this seems to be the best and shortest way of presenting an idea of the scope and character of this excellent quarterly production.

